# Enhancement of Carrier Mobility in Multilayer InSe Transistors by van der Waals Integration

**DOI:** 10.3390/nano14040382

**Published:** 2024-02-19

**Authors:** Zhiwei Li, Jidong Liu, Haohui Ou, Yutao Hu, Jiaqi Zhu, Jiarui Huang, Haolin Liu, Yudi Tu, Dianyu Qi, Qiaoyan Hao, Wenjing Zhang

**Affiliations:** 1 State Key Laboratory of Radio Frequency Heterogeneous Integration, Shenzhen University, Shenzhen 518060, China; 2200493031@email.szu.edu.cn (Z.L.); ljd@szu.edu.cn (J.L.); ouhaohui2016@email.szu.edu.cn (H.O.); huyutao2022@email.szu.edu.cn (Y.H.); jqzhu@email.szu.edu.cn (J.Z.); 2110496016@email.szu.edu.cn (J.H.); 2310492039@email.szu.edu.cn (H.L.); tu.yudi@szu.edu.cn (Y.T.); 2 International Collaborative Laboratory of 2D Materials for Optoelectronics Science and Technology of Ministry of Education, Institute of Microscale Optoelectronics, Shenzhen University, Shenzhen 518060, China; 3 Zhejiang Technology Innovation Center of CMOS IC Manufacturing Process and Design, College of Integrated Circuits, Zhejiang University, Hangzhou 311200, China; qidianyu@zju.edu.cn

**Keywords:** field effect transistor, 2D materials, van der Waals integration, carrier mobility

## Abstract

Two-dimensional material indium selenide (InSe) holds great promise for applications in electronics and optoelectronics by virtue of its fascinating properties. However, most multilayer InSe-based transistors suffer from extrinsic scattering effects from interface disorders and the environment, which cause carrier mobility and density fluctuations and hinder their practical application. In this work, we employ the non-destructive method of van der Waals (vdW) integration to improve the electron mobility of back-gated multilayer InSe FETs. After introducing the hexagonal boron nitride (h-BN) as both an encapsulation layer and back-gate dielectric with the vdW interface, as well as graphene serving as a buffer contact layer, the electron mobilities of InSe FETs are substantially enhanced. The vdW-integrated devices exhibit a high electron mobility exceeding 10^3^ cm^2^ V^−1^ s^−1^ and current on/off ratios of ~10^8^ at room temperature. Meanwhile, the electron densities are found to exceed 10^12^ cm^−2^. In addition, the fabricated devices show an excellent stability with a negligible electrical degradation after storage in ambient conditions for one month. Electrical transport measurements on InSe FETs in different configurations suggest that a performance enhancement with vdW integration should arise from a sufficient screening effect on the interface impurities and an effective passivation of the air-sensitive surface.

## 1. Introduction

Two-dimensional semiconductors (2DSM) have been widely employed as field effect transistor (FET) channel materials due to their tunable energy band structures and excellent electronic properties [1,2]. With dangling-bond-free surfaces and atomically uniform thicknesses, they hold great promise for replacing conventional silicon-based devices to overcome the performance limitations caused by short channel effects. Moreover, the transport behaviors of 2DSM could be manipulated through creating van der Waals (vdW) heterostructures without strict lattice matching or processing compatibility [3,4,5]. Unlike bulk materials, the performances of 2DSM-based devices are controlled and determined by the surface and interface characteristics of the channel, which typically involve the semiconductor surface, 2DSM-metal contact, and 2DSM–dielectric interface [6,7,8]. First, the surface effect in a 2DSM transistor mainly stems from the interaction between the surface of the channel and active ingredients in air, resulting in the modification of fundamental properties for sensitive 2DSM [9]. Second, the non-ideal 2DSM-metal contact is one of the major performance-limiting factors for 2DSM transistors, which arises from the gap states and interface disorders induced by the lithography and deposition processes and leads to the Fermi-level pinning (FLP) effect at the contact interface [10,11]. Third, the 2DSM–dielectric interface, especially for conventional dielectrics such as SiO_2_ and Al_2_O_3_, is responsible for the lowered carrier mobility due to the charged surface states, impurities, and surface roughness [12]. So far, extensive efforts have been made to improve the channel properties and push the performance limit of transistors by using various strategies, including contact optimization [13,14], reduction of impurity scattering [15], dielectric engineering [16,17,18], atomic vacancy healing and so on [19,20].

2D indium selenide (InSe) is a highly promising material for future nanoelectronics in virtue of several unique characteristics, such as a small effective electron mass, good charge transport behavior, and extraordinary mechanical properties [21,22]. Recent studies have shown that thin InSe layers exhibit spontaneous surface oxidation upon exposure to ambient atmosphere, forming charged traps at the surface and remarkable current hysteresis in transistors [23,24]. To address the reliability issue, various encapsulation approaches have been explored and proven to be effective in suppressing the infiltration of moisture and oxygen [25]. On the other hand, the charge transport of InSe transistors is sensitive to the dielectric environment. It has been revealed that the electron mobility of InSe nanosheets can be greatly improved by modulating the types of dielectric materials due to the strong suppression of carrier scattering [26,27]. In particular, integrating 2DSM with different dielectrics via vdW interactions provides vast opportunities for probing the intrinsic properties of 2DSM by creating high-quality interfaces with a low interfacial state density [28,29]. Despite the rapid progress in boosting the carrier mobility of InSe channels, its surface and interface issues must still be effectively addressed to push the development of high-performance InSe FETs [30,31].

In this study, we aim to improve the charge carrier mobility of InSe transistors by a non-destructive vdW integration process. In this approach, a hybrid gate dielectric, composed of a hexagonal boron nitride (h-BN) layer and subjacent SiO_2_ layer, is used to engineer the 2DSM–dielectric interface, a graphene buffer layer serves as electrical contacts to the InSe channel, and a top h-BN encapsulation layer is integrated to isolate the InSe layer from ambient conditions and achieve the long-term stability of the devices. This strategy enables us to gain a high electron mobility of up to 1078 cm^2^ V^−1^ s^−1^ at room temperature and an on/off ratio of up to ~10^8^, which is larger than that of transistors based on the bare SiO_2_ dielectric and hybrid gate dielectric of polymer and Al_2_O_3_. Moreover, optoelectronic measurements in the multilayer InSe transistors show an excellent photoresponse with a high photoresponsivity and external quantum efficiency (EQE).

## 2. Materials and Methods

### 2.1. Fabrication of Multilayer InSe Transistors

Bulk h-BN and graphene crystals were purchased from HQ graphene. Bulk InSe crystals were synthesized from melting In powders and Se powders in a sealed vacuum tube inside a furnace. The sealed tube was heated to 700 °C for 3 h and kept at 700 °C for 3 days. After that, it was cooled to 500 °C for 2 days, followed by natural cooling. The back-gated FETs were fabricated using a layer-by-layer dry transfer method in an argon-filled glove box (H_2_O < 1 ppm, O_2_ < 1 ppm). First, the h-BN crystals were mechanically exfoliated using blue tape onto the polydimethylsiloxane (PDMS) and then transferred onto an oxidized silicon wafer, which was pre-processed by ultraviolet-ozone cleaning for 5 min. The silicon wafer with h-BN flakes was annealed on a hot stage in the glove box at 400 °C for 3 h. Then, two few-layer graphene flakes were transferred onto two ends of the bottom h-BN to serve as the buffer layer. The h-BN/graphene heterostructure was also annealed in the glove box at 400 °C for 3 h to remove processing residues. After that, the target InSe flakes on PDMS were aligned on the h-BN/graphene heterostructure so that the edges of the InSe flakes overlapped with the graphene layer. The h-BN/graphene/InSe heterostructure was annealed at 180 °C for 5 min. Finally, a larger h-BN flake was selected to encapsulate the heterostructure, followed by another annealing at 180 °C for 5 min. The metal electrodes of Cr/Au (10/50 nm) were patterned using a copper grid as the shadow mask and prepared by standard electron-beam evaporation (ASB-EPI-C6, Syskey Technology Co., Ltd., Hsinchu, Taiwan). The fabricated devices were annealed in an Ar atmosphere at 180 °C for 30 min to optimize the contact condition.

For the FETs devices with PMMA/Al_2_O_3_ bilayer dielectric, the 30 nm thick Al_2_O_3_ was grown by atomic layer deposition (ALD) System MNT-S100-L3S1 (MNT Micro and Nanotech Co., Ltd., Wuxi, China). The upper PMMA layer was fabricated by spin-coating (2000 rpm, 2 min) 2.5 wt% 950k PMMA on Al_2_O_3_/Si substrate and baked at 170 °C for 30 min.

### 2.2. Characterization and Device Measurement

The topographies and thicknesses of exfoliated nanosheets (InSe, graphene, h-BN) and films of PMMA and Al_2_O_3_ were identified by an optical microscope (Nikon Y-IDP) and AFM (Dimension ICON system, Bruker, Billerica, MA, USA). Raman and PL spectra of InSe nanosheets were collected by using a WITec Alpha 300R (Oxford Instruments, Abingdon, UK) confocal microscope spectrometer with an excitation laser of 532 nm. TEM sample was prepared by using the focused ion beam (FIB) system (FEI, Scios, Hopewell Township, NJ, USA). To protect the selected region from ion damage, a layer of 500 nm Pt and another layer of 2 μm Pt were successively deposited on the h-BN/InSe/h-BN heterostructure before FIB milling. The prepared cross-sectional slice was then transferred and attached to a TEM grid. TEM and EDS mapping images were performed on a JEOL JEM-3200FS operated at an accelerating voltage of 300 kV and equipped with an EDS detector.

The electrical properties of the InSe devices were measured in a probe station (Lakeshore, TTPX, Thebarton, Australia) in vacuum, using a Keithley (4200-SCS, Cleveland, OH, USA) semiconductor parameter analyzer. Photo-response was triggered by a supercontinuum white-light laser source (SC400-8, Fianium Ltd., Southampton, UK) coupled with a monochromator. The laser intensity was measured with a commercial Thorlabs power meter.

## 3. Results and Discussion

**Figure 1:**
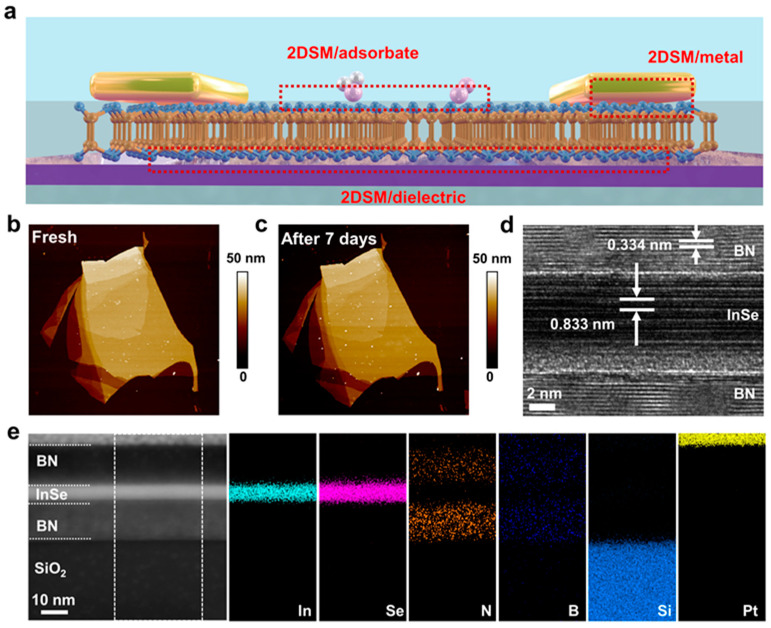
(**a**) Schematic of the three types of interfaces in the InSe FET device, including InSe/adsorbate, InSe/metal contact, and InSe/dielectric interfaces, as highlighted by the dotted boxes. (**b**) AFM image of a fresh InSe nanosheet prepared by mechanical cleavage. (**c**) AFM image of the InSe nanosheet after seven days of ambient exposure. (**d**) Cross-sectional HRTEM image of h-BN/InSe/h-BN vdW heterostructure, showing good interface quality. (**e**) EDS element mappings of the h-BN/InSe/h-BN heterostructure.

As illustrated in Figure 1a, there are three types of interfaces involved in the InSe FET device, including the InSe/adsorbate interface, InSe/metal contact, and InSe/dielectric interface. Figure 1b shows the atomic force microscopy (AFM) image of a 20 nm thick InSe nanosheet shortly after exfoliation, showing a uniform topography. After seven days of ambient exposure, the inspection of increased small protrusions on the surface suggested structural or chemical changes in the InSe nanosheet arising from the slow but spontaneous oxidation (Figure 1c) [23]. Moreover, the photoluminescent (PL) emission showed an appreciable reduction in intensity (Figure S1). Accordingly, the vdW integration method was employed to achieve pure van der Waal contacts and minimize defects and charge trapping sites by providing a high interface quality for optoelectronic devices [32]. In the realm of vdW integration, artificial heterostructures composed of 2DSM, graphene, and h-BN have gained extensive attention [28,33]. For the sake of simplicity, we fabricated vdW heterostructures composed of a multilayer InSe layer interlaid between thin h-BN layers using a dry alignment transfer procedure shown in Figure S2. High-resolution transmission electron microscopy (HRTEM) for the interface region was used to characterize the vdW interface region (Figure 1d). Obviously, the h-BN/InSe/h-BN stacking could produce damage-free and atomically smooth interfaces between disparate materials. The interlayer spacing was measured to be 0.334 nm and 0.833 nm, equal to the interlayer distance in h-BN and InSe, respectively. The corresponding energy-dispersive X-ray spectroscopy (EDS) element mappings in Figure 1e distinctly presented the individual components of the h-BN/InSe/h-BN heterostructure, including the deposited Pt layer, InSe layer, and top and bottom h-BN layers.

**Figure 2:**
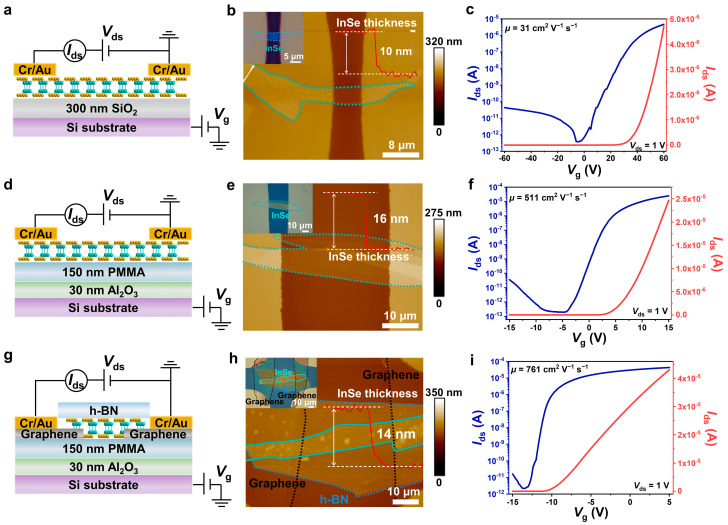
(**a**,**b**) Schematic view and AFM image of the InSe FET based on bare SiO_2_ dielectric. Inset shows the corresponding optical image of the device. (**c**) Transfer characteristics of the device at *V*_ds_ = 1 V at linear (red line) and logarithmic (blue line) scales. (**d**,**e**) Schematic view and AFM image of the InSe FET based on PMMA/Al_2_O_3_ dielectric without encapsulation. (**f**) Transfer characteristics of the device at *V*_ds_ = 1 V at linear (red line) and logarithmic (blue line) scales. (**g**,**h**) Schematic view and AFM image of the h-BN-encapsulated InSe FET based on PMMA/Al_2_O_3_ dielectric. (**i**) Transfer characteristics of the device at *V*_ds_ = 1 V at linear (red line) and logarithmic (blue line) scales.

Apart from the surface structure, the interface between the InSe and dielectric layer is one of the crucial factors dominating the FET device performance. To this end, we fabricated different types of back-gated field effect transistor (FET) devices based on various dielectric layers using the home-made copper grid mask method. The InSe nanosheets with a thickness range of 10–30 nm were exfoliated from bulk crystals and transferred to silicon substrates. Figure 2a illustrates the schematic view of the InSe/SiO_2_ FET device based on a single 300 nm thick SiO_2_ dielectric. The thickness of the InSe channel was 10 nm, as measured by AFM in Figure 2b. The transfer and output characteristics of InSe/SiO_2_ FET were carried out by applying a drain-source voltage (*V*_ds_) and gate voltage (*V*_g_) in a three-terminal configuration. Figure 2c presents the *I*_ds_-*V*_g_ curves of the typical InSe/SiO_2_ FET operated at *V*_ds_ = 1 V, indicating an obvious n-type behavior with a current on/off ratio of 1.3 × 10^7^. The field-effect mobility can be extracted using the equation as follows:

*μ* = (d*I*_ds_/d*V*_g_) × *L*/(*WC*_i_*V*_ds_) (1)

where *L* and *W* are the channel length and width of the device; and *C*_i_ is the capacitance between the channel and the back gate per unit area: *C*_i_ = *ε*_0_*ε*_r_/*d*, where *ε*_0_ = 8.85 × 10^−12^ F m^−1^ and *ε*_r_ (3.9) and *d* (300 nm) are the dielectric constant and thickness of SiO_2_, respectively. Based on the transport curve, the electron mobility was calculated to be 31 cm^2^ V^−1^ s^−1^. The carrier scattering originating from the amorphous gate dielectric of SiO_2_ was primarily responsible for the low electron mobility [27,28]. The output curves of the InSe/SiO_2_ FET showed that a larger positive gate voltage induced a higher current due to the n-type conduction (Figure S3). To screen the interfacial Coulomb impurities, we also fabricated an InSe FET device based on a hybrid dielectric interface, composed of 150 nm poly(methyl methacrylate) (PMMA) and 30 nm Al_2_O_3_ (Figure 2d). The optical micrograph and AFM images of InSe/PMMA/Al_2_O_3_ FET are shown in Figure 2e. Figure 2f presents the *I*_ds_-*V*_g_ curves of the typical InSe/PMMA/Al_2_O_3_ device, demonstrating an electron mobility of 511 cm^2^ V^−1^ s^−1^ and on/off ratio of ~10^8^, respectively. The carrier mobility was one order of magnitude higher than that from the FET device on the SiO_2_/Si substrate. Furthermore, multilayer InSe was encapsulated with h-BN to prevent absorbed impurities and oxidations on the channel surface. Figure 2g illustrates the schematic view of the h-BN/InSe/PMMA/Al_2_O_3_ FET device, with graphene serving as the buffer layer. The thickness of the InSe channel was identified to be 14 nm according to the AFM in Figure 2h. The mobility of h-BN/InSe/PMMA/Al_2_O_3_ FET was further enhanced to be 761 cm^2^ V^−1^ s^−1^ with an on/off ratio of 2.1 × 10^7^ (Figure 2i).

**Figure 3:**
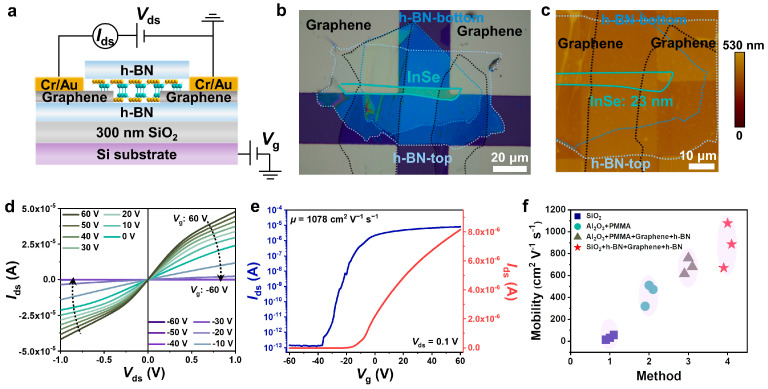
(**a**) Schematic view of the h-BN-encapsulated InSe FET based on h-BN/SiO_2_ dielectric. (**b**,**c**) Optical image and the corresponding AFM image of the vdW-integrated h-BN/InSe/h-BN/SiO_2_ FET device. (**d**) Output characteristics of the device at different *V*_g_. (**e**) Transfer characteristics of the device at *V*_ds_ = 0.1 V at linear (red line) and logarithmic (blue line) scales. (**f**) The distribution of electron mobility for InSe devices with different configurations.

Recently, h-BN has been widely explored to optimize interface properties and electrical performance because of its high single crystallinity and vdW surfaces. A root-mean-square surface roughness of 0.19 nm was measured for the cleaved h-BN (Figure S4), which was almost the same as for the defect-free polymer of PMMA (0.29 nm). Hence, h-BN was further employed as both a gate dielectric and passivation layer to fabricate InSe FET devices using the vdW integration process. In addition, graphene nanosheets were used as a buffer layer to create a high-quality electrical contact and alleviate the damage of the InSe channel during the metal deposition process [34]. Details of the device fabrication process were described in the Materials and Methods section and Figure S5. Figure 3a,b display the schematic view of h-BN/InSe/h-BN/SiO_2_ FET and optical images of a typical device, respectively. The thickness of the InSe channel was determined to be 23 nm by AFM measurement (Figure 3c and Figure S6). Note that the channel was fully covered by a top h-BN layer, as marked by the light blue dotted line. Figure 3d shows the *I*_ds_-*V*_ds_ curves for back gate voltages ranging from −60 to +60 V, indicating the good contact between the Cr/Au electrodes and InSe channel. The satisfactory performance should be attributed to the formation of a vdW contact with the insertion of an atomically flat graphene layer, which is efficient in preventing metal diffusion from electrodes to the channel and eliminating the metal-induced gap state [10]. Accordingly, the FLP effect between the InSe and Au electrode was alleviated, and an enhanced carrier mobility could be expected. Figure 3e shows the transfer curves of the h-BN/InSe/h-BN/SiO_2_ FET, resulting in a high electron mobility of 1078 cm^2^ V^−1^ s^−1^ and an on/off ratio of 7.1 × 10^7^ at room temperature. This mobility value was equivalent to the previous results from the top- or bottom-gated FET devices reported for multilayer InSe, which were optimized by interface engineering methods, including encapsulation, modifying the contact metal or dielectric layer, and surface charge doping [21,28,30]. The hysteresis behavior in the vdW-integrated InSe was also revealed. As shown in Figure S7, the device exhibited a diminutive hysteresis loop due to the decrease of interfacial trap densities, consistent with a previous study [12]. The carrier density (*n*) was calculated to be 2.5 × 10^12^ cm^−2^ according to the following equation [35], where *q* is the elemental charge:

*n* = (*I*_ds_*L*)/(*qWV*_ds_*μ*) (2)

Moreover, Figure 3f and Figure S8 summarized the distribution of electron mobility (*μ*) for four types of multilayer InSe FET devices fabricated in different batches, as marked by square, circle, triangle, and star, respectively. Overall, the vdW-integrated InSe FETs exhibited a substantial improvement in carrier mobility compared to the case involving an InSe channel placed directly on the SiO_2_/Si substrate and also devices without encapsulation. Furthermore, the mobility of vdW-integrated devices was competitive with that of devices based on a PMMA dielectric layer, suggesting the effective screening of carrier scattering by h-BN. In order to see environmental stability, the evolution of the electrical properties of the devices was further measured. It turned out that the vdW-integrated devices could maintain a stable carrier mobility and current on/off ratio when they were stored under ambient conditions for one month (Figure S9). These experimental results indicate that the clean interface of the 2DSM/dielectric and the h-BN-passivated surface of the 2DSM channel contribute to the preservation of charge transport.

**Figure 4:**
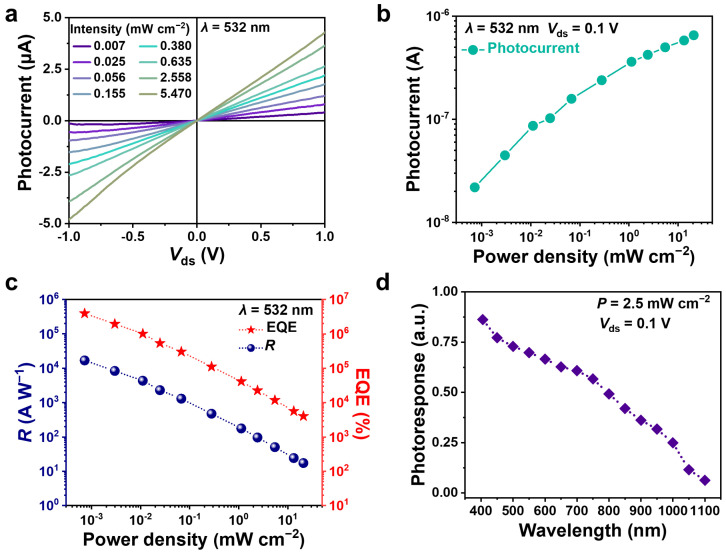
(**a**) *I*_ph_ − *V*_ds_ curves of the vdW-integrated InSe device under different 532 nm laser power densities. (**b**) Dependence of the photocurrent on the power density at *V*_ds_ = 0.1 V. (**c**) Dependence of the photoresponsivity and EQE on the power density. (**d**) Wavelength-dependent photoresponsivity under incident light illumination with a fixed power density of 2.5 mW cm^−2^.

Furthermore, the photoresponse performance of the vdW-integrated InSe transistors was investigated. Under illumination of a 532 nm laser, the InSe device showed linear source-drain current-voltage (*I*_ds_ − *V*_ds_) curves (Figure S10), indicating good electrical contact between the channel and metal electrodes. Furthermore, the photocurrent (*I*_ph_), defined as the difference between the device current measured under laser light illumination (*I*_light_) and that measured in the dark (*I*_dark_), increased monotonically when increasing the laser power density (*P*) of the incident laser light, as displayed in Figure 4a,b. To evaluate the photodetector performance, the photoresponsivity (*R*) and external quantum efficiency (EQE) were calculated according to the following equations:

*R* = *I*_ph_/(*PS*) (3)

*EQE* = *R*/(*hc/eλ*) (4)

where *S* is the effective area of the device, *h* is the Planck constant, and *c* is the speed of light. Figure 4c plots the value of photoresponsivity under different power densities. When the photodetector operated at *V*_ds_ = 0.1 V and *V*_g_ = 0 V, a maximum photoresponsivity of 1.7 × 10^4^ A W^−1^ was obtained at a low power density of 7.2 × 10^−4^ mW cm^−2^. Meanwhile, EQE showed a similar trend to photoresponsivity when the power densities increased, resulting in a maximum value of 3.9 × 10^6^% at *P* = 7.2 × 10^−4^ mW cm^−2^. Figure 4d shows the wavelength-dependent photoresponsivity of the InSe device measured at a fixed laser power density of 2.5 mW cm^−2^ (*V*_ds_ = 0.1 V, *V*_g_ = 0 V). When the photo energy was larger than the band gap (1.26 eV) of multilayer InSe, abundant electron–hole pairs would be excited and subsequently separated by the bias voltage, leading to an efficient photoresponse in the wavelength range of 400–950 nm. When the wavelength increased towards 1100 nm, the devices exhibited an obvious decrease in photoresponsivity due to the forbidden interband absorption, in agreement with a previous study [36].

## 4. Conclusions

In summary, we have demonstrated the drastic effect of interfacial and surface properties on the electrical properties of multilayer InSe by fabricating FET devices with different configurations. Taking advantage of the vdW integration technique, the h-BN/InSe/h-BN/SiO_2_ FET displayed room-temperature electron mobility exceeding 10^3^ cm^2^ V^−1^ s^−1^ with an on/off ratio of 7.1 × 10^7^ and electron density of 2.5 × 10^12^ cm^−2^, respectively, which was an order of magnitude larger than that from FETs based on an oxide dielectric. Such an enhanced device performance was mainly due to a clean InSe/dielectric interface and vdW contact between the InSe channel and metal electrodes with the insertion of a graphene buffer layer. Moreover, the passivated surface of the InSe channel enabled an excellent environmental stability of the transistors. This work demonstrates that vdW integration of 2DSM is promising for realizing high-performance electronic devices.

## Data Availability

Data are contained within the article and the Supplementary Material.

## Supplementary Materials

The following supporting information can be downloaded at: https://www.mdpi.com/article/10.3390/nano14040382/s1, Figure S1. Evolution of PL spectra for the exfoliated InSe nanosheet after seven days of ambient exposure. Figure S2. Optical image of the fabricated h-BN/InSe/h-BN heterostructure for characterization of the vdW interface. Figure S3. Output characteristics of (a) InSe/SiO_2_ FET, (b) InSe/PMMA/Al_2_O_3_ FET, and (c) h-BN/InSe/PMMA/Al_2_O_3_ FET devices at different *V*_g_. Figure S4. 3D surface images of the cleaved h-BN and spin-coated PMMA film, with a root-mean-square surface roughness of 0.19 and 0.29 nm, respectively. Figure S5. Dry transfer process to fabricate a h-BN/InSe/h-BN/SiO_2_ vdW heterostructure with graphene as the buffer layer. Scale bars, 20 μm. Figure S6. Height profile extracted from InSe layer of the vdW-integrated heterostructure, showing a channel thickness of 23 nm. Figure S7. Transfer curves of the vdW-integrated FET with forward and backward scans of the gate voltages. Figure S8. Transfer characteristics at linear scales of (a) InSe/SiO_2_ FET, (b) InSe/330 nm PMMA/30 nm Al_2_O_3_ FET, (c) h-BN/InSe/150 nm PMMA/30 nm Al_2_O_3_ (up) and h-BN/InSe/330 nm PMMA/30 nm Al_2_O_3_ (bottom) FET, and (d) h-BN/InSe/h-BN/SiO_2_ FET devices, respectively. Figure S9. Comparison of transfer curves of the vdW-integrated FET after one month exposure to ambient atmosphere, revealing the excellent stability of the device. Figure S10. *I*_ds_ − *V*_ds_ curves of the vdW-integrated InSe device under dark conditions and different 532 nm laser power densities.
