# Supplementary material for: Enhancement of Carrier Mobility in Multilayer InSe Transistors by van der Waals Integration
Source: Nanomaterials (Basel). 2024 Feb 19;14(4):382. doi: 10.3390/nano14040382 (PMC10892934; doi:10.3390/nano14040382)
Supplement: Supplementary file 1 [file nanomaterials-14-00382-s001.zip › nanomaterials-2871283-supplementary.pdf]

nanomaterials

Supplementary Materials

Enhancement of Carrier Mobility in Multilayer InSe Transistors by van der Waals Integration

Zhiwei Li [1,2,†], Jidong Liu [1,2,†], Haohui Ou [1,2], Yutao Hu [1,2], Jiaqi Zhu [1,2], Jiarui Huang [1,2], Haolin Liu [1,2], Yudi Tu [1,2], Dianyu Qi [3], Qiaoyan Hao [1,2,*] and Wenjing Zhang [1,2,*]

[1] State Key Laboratory of Radio Frequency Heterogeneous Integration, Shenzhen University, Shenzhen 518060, China; 2200493031@email.szu.edu.cn (Z.L.); ljd@szu.edu.cn (J.L.); ouhaohui2016@email.szu.edu.cn (H.O.); huyutao2022@email.szu.edu.cn (Y.H.); jqzhu@email.szu.edu.cn (J.Z.); 2110496016@email.szu.edu.cn (J.H.); 2310492039@email.szu.edu.cn (H.L.); tu.yudi@szu.edu.cn (Y.T.)

[2] International Collaborative Laboratory of 2D Materials for Optoelectronics Science and Technology of Ministry of Education, Institute of Microscale Optoelectronics, Shenzhen University, Shenzhen 518060, China

[3] Zhejiang Technology Innovation Center of CMOS IC Manufacturing Process and Design, College of Integrated Circuits, Zhejiang University, Hangzhou 311200, China; qidianyu@zju.edu.cn

* Correspondence: hqy@szu.edu.cn (Q.H.); wjzhang@szu.edu.cn (W.Z.)

† These authors contributed equally to this work.

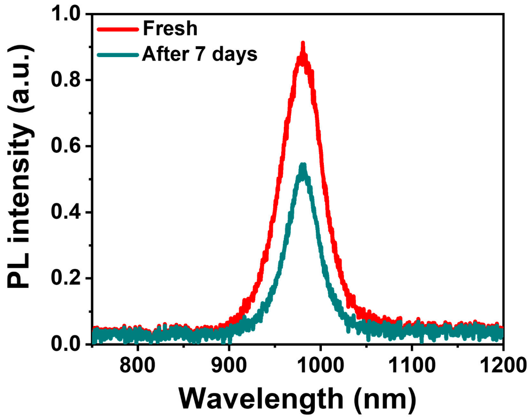

Figure S1. Evolution of PL spectra for the exfoliated InSe nanosheet after seven days of ambient exposure.

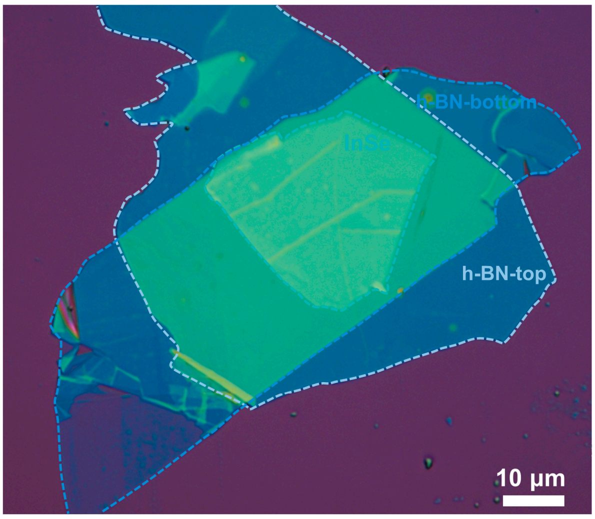

Figure S2. Optical image of the fabricated h-BN/InSe/h-BN heterostructure for characterization of the vdW interface.

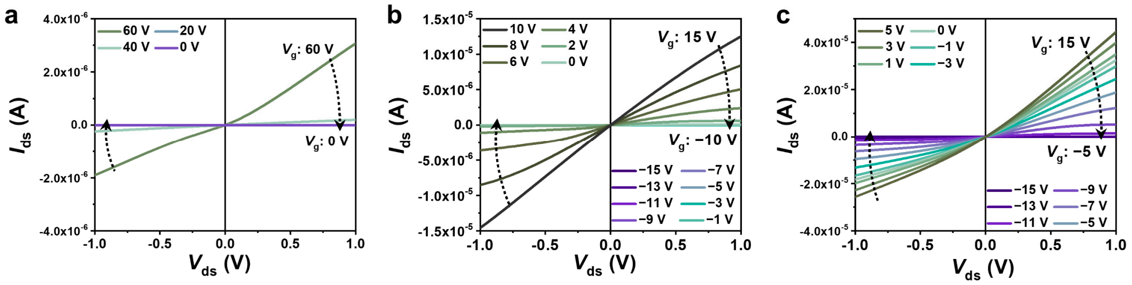

Figure S3. Output characteristics of (a) InSe/SiO_2 FET, (b) InSe/PMMA/Al_2O_3 FET, and (c) h-BN/InSe/PMMA/Al_2O_3 FET devices at different V_g.

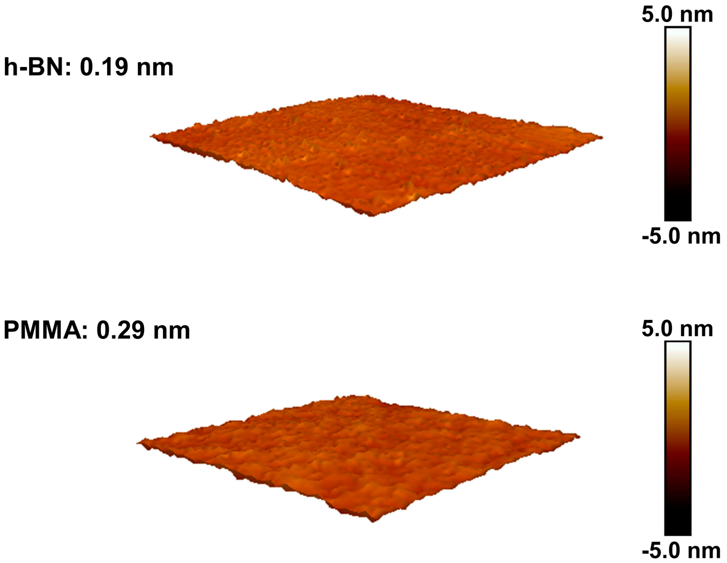

Figure S4. 3D surface images of the cleaved h-BN and spin-coated PMMA film, with a root-mean-square surface roughness of 0.19 and 0.29 nm, respectively.

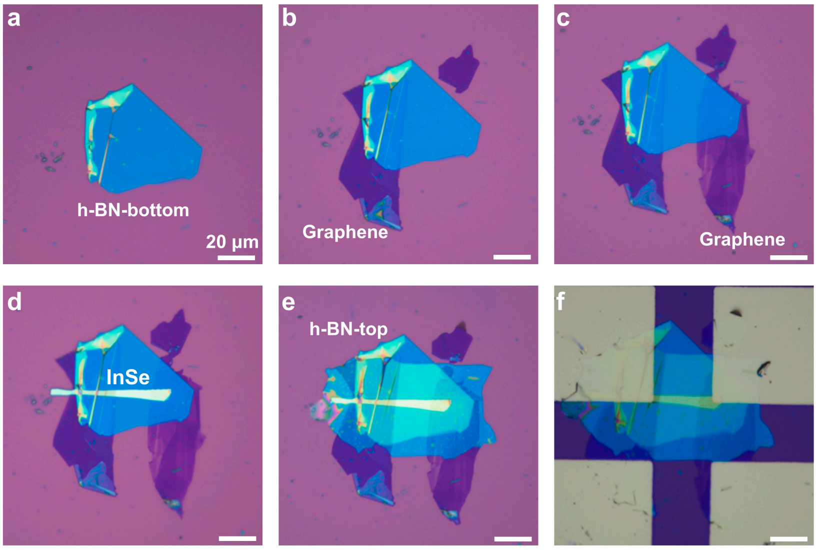

Figure S5. Dry transfer process to fabricate the h-BN/InSe/h-BN/SiO_2 heterostructure with graphene as the buffer layer. Scale bars, 20 μm.

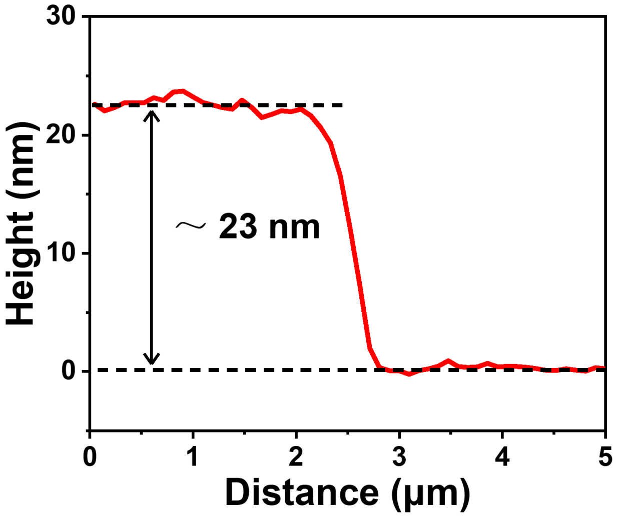

Figure S6. Height profile extracted from InSe layer of the vdW integrated heterostructure, showing the channel thickness of 23 nm.

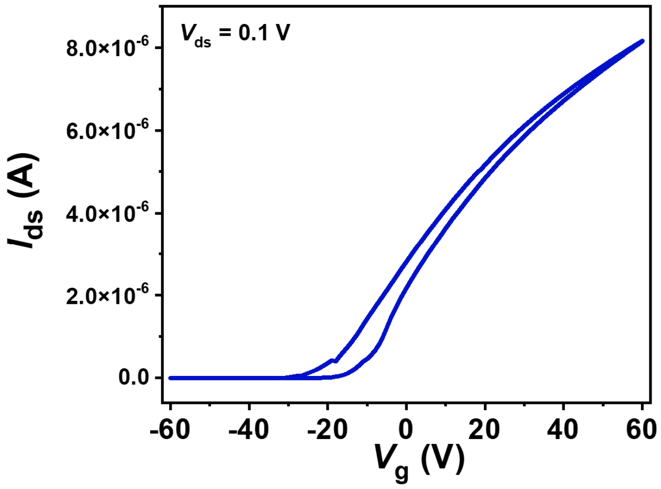

Figure S7. Transfer curves of the vdW integrated FET with forward and backward scans of the gate voltages.

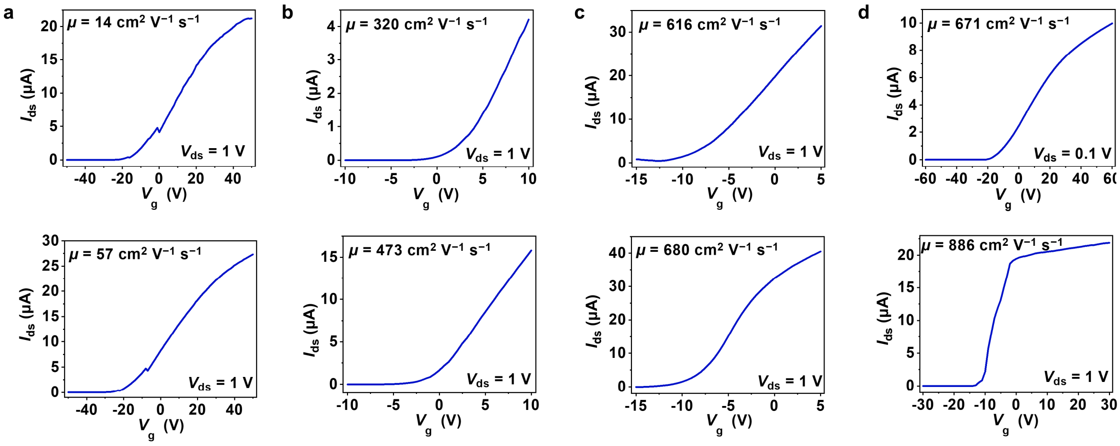

Figure S8. Transfer characteristics in linear scales of (a) InSe/SiO_2 FET, (b) InSe/330 nm PMMA/30 nm Al_2O_3 FET, (c) h-BN/InSe/150 nm PMMA/30 nm Al_2O_3 (up) and h-BN/InSe/330 nm PMMA/ 30 nm Al_2O_3 (bottom) FET, and (d) h-BN/InSe/h-BN/SiO_2 FET devices, respectively.

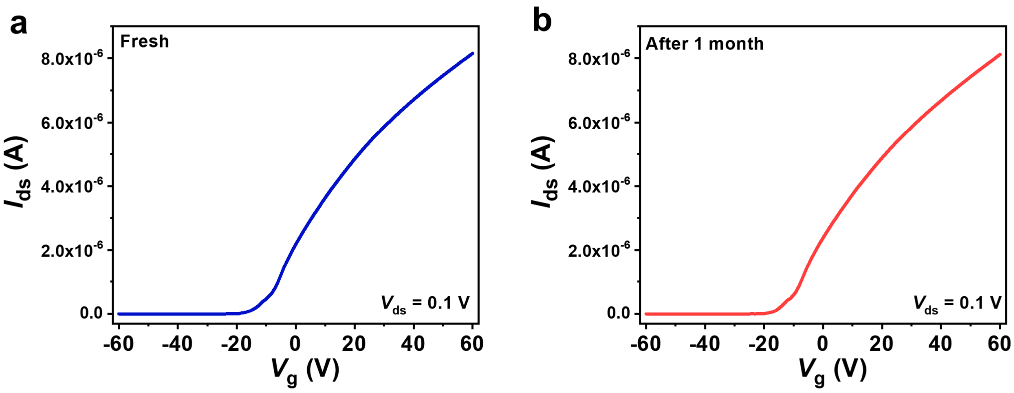

Figure S9. Comparison of transfer curves of the vdW integrated FET upon one month' exposure to ambient atmosphere, revealing the excellent stability of the device.

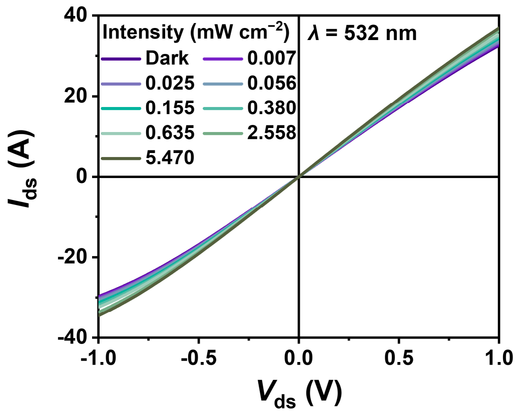

Figure S10. I_{ds}–V_{ds} curves of the vdW integrated InSe device under dark and different 532 nm laser power densities.
